# Preventing synaptic deficits in Alzheimer’s disease by inhibiting tumor necrosis factor alpha signaling

**DOI:** 10.1016/j.ibror.2018.01.003

**Published:** 2018-02-02

**Authors:** Chelsea Cavanagh, Tak Pan Wong

**Affiliations:** a Douglas Mental Health University Institute, Montreal, QC, Canada; b Department of Psychiatry, McGill University, Montreal, QC, Canada

**Keywords:** Amyloid precursor protein, Hippocampus, Neuroinflammation, Prevention, TgCRND8 mice, Tumor necrosis factor alpha, XPro1595

## Abstract

The characterization of preclinical stages of Alzheimer’s disease (AD) would provide a therapeutic window for prevention. One of the challenges of developing preventive therapy for AD is to identify early biomarkers for intervention studies. We have recently shown that in the TgCRND8 transgenic AD mouse model, increased hippocampal levels of the pro-inflammatory cytokine tumor necrosis factor alpha (TNFα) and enhanced excitatory synaptic transmission were early-onset changes that occurred weeks before amyloid plaque formation. Inhibiting TNFα before plaque formation not only normalized excitatory synaptic function, but also prevented the impairment of synaptic function 4 months later. In this review paper, we will examine the potential contributions of TNFα to the alteration of brain function in preclinical AD. The prospective use of TNFα inhibitors for preventing AD will be discussed.

Alzheimer’s disease (AD) is a neurodegenerative disorder and the most common cause of dementia, which affects close to 50 million people worldwide (AD-International, 2015). While the prevalence of AD is expected to be tripled by the mid-21st century, effective AD treatment is lacking. Findings from at-risk genetic cohorts or older asymptomatic people have revealed a long pathophysiological process that starts years, if not decades, before the onset of clinical AD. Several preclinical AD stages before the onset of overt cognitive decline have been proposed and are currently characterized by studying changes in imaging modalities and biomarkers such as amyloid and tau levels in cerebrospinal fluid (Sperling et al., 2011). While the accumulated brain damage in patients with AD dementia is likely to be irreversible, preclinical AD could be a time window for preventive treatment. Indeed, while increased AD risk has been associated with mid-life factors, such as hypertension, physical inactivity and poor education (Barnes and Yaffe, 2011), clinical trials have revealed some protective effects of antihypertensive therapy, exercise, and cognitive training (Andrieu et al., 2015). An effective preventive treatment could have a significant impact on the economic burden of AD to society, since delaying the onset age of AD by 5 years has been estimated to reduce the incidence of AD by 50% (Brookmeyer et al., 1998). A challenge for developing a preventive treatment for AD is to characterize and validate earlier biomarkers for intervention studies.

Neuroinflammation could be an early stage biomarker for preclinical AD. While the idea that anti-inflammatory treatment underlies the low prevalence of AD in rheumatic arthritis patients has been around for over 25 years (McGeer et al., 1990), the role of neuroinflammation remains unclear in the transition from asymptomatic preclinical stages to AD dementia. Pro-inflammatory agents like cytokines have been implicated in the pathophysiology of AD. For example, interferon-γ (IFN-γ) mRNA expression is elevated in the Tg2576 mouse model of AD at early stages of amyloid pathology (Abbas et al., 2002). In addition, TNFα along with monocyte chemoattractant protein were also shown to be increased before amyloid plaque deposition in the entorhinal cortex of 3xTgAD mice, another mouse model of AD (Janelsins et al., 2005). Evidence of neuroinflammation is also present in patients with mild cognitive impairment who have increased caspase-1 (Heneka et al., 2013). The TgCRND8 mouse model that expresses the human amyloid precursor protein (APP) gene with both the Indiana (V717F) and Swedish (K670N/M671L) mutations, which leads to an overproduction of amyloid-β (Aβ). Using this model, we found that the levels of the pro-inflammatory cytokine, tumor necrosis factor α (TNFα) rise in 1-month old mice, 2 months before the onset of amyloid pathology and cognitive impairment (Cavanagh et al., 2013), which becomes prevalent at 3 months of age in this model (Chishti et al., 2001). This early-stage increase in TNFα levels can have a significant impact on brain functions. TNFα enhances neuronal excitability by inserting the AMPA subtype of glutamate receptors and removing GABA_A_ receptors from synapses (Stellwagen and Malenka, 2006). Notably, enhanced hippocampal activation has been observed in cognitively normal carriers of apolipoprotein E4 (ApoE4) (Nichols et al., 2012) or presenilin 1 (PS1) gene mutations (Reiman et al., 2012), and these individuals also have an increased risk of developing dementia. Enhanced hippocampal activation may reflect an increase in activity at the synaptic level. Therefore, we hypothesize that increased TNFα signaling at an early stage may lead to synaptic hyperexcitability, which could precipitate synaptic and cognitive pathology in AD.

In a recent study (Cavanagh et al., 2016), we examined the contribution of enhanced levels of TNFα on functional properties of glutamate synapses in the hippocampus of pre-plaque (1-month old) TgCRND8 mice. We observed stronger glutamatergic transmission in the hippocampus of pre-plaque TgCRND8 mice than in control, nontransgenic littermates. At this young age, TgCRND8 mice also exhibited higher hippocampal synaptic (long-term potentiation) and cognitive function (passive avoidance) than control mice (Cavanagh et al., 2016). Moreover, enhanced hippocampal synaptic and cognitive functions were normalized by the TNFα inhibitor, XPro1595 (Cavanagh et al., 2016). This novel biologic is a dominant-negative version of TNFα that can form inactive trimers with endogenous soluble TNFα to thereby prevent TNFα receptor 1 (TNFR1) activation (Steed et al., 2003), which mediates signaling that is often pathogenic and can lead to cell death (Van Hauwermeiren et al., 2011). These findings support a role of TNFα in enhancing hippocampal synaptic function before amyloid plaque formation. To determine the impact of the early-stage increase in TNFα on the formation of synaptic impairments that have been observed in post-plaque 6-12 month old TgCRND8 mice (Kimura et al., 2012), we administered XPro1595 in 1-month old TgCRND8 mice through a subcutaneously implanted osmotic pump for 4 weeks. After pump removal, mice were left undisturbed until they became 6-month old. Compared with control mice, 6-month old TgCRND8 mice exhibited a decrease in glutamatergic transmission in the hippocampus. Decreased synaptic function however was rescued in XPro1595-treated TgCRND8 mice (Cavanagh et al., 2016). To our knowledge, this was the first study to show that inhibiting TNFα signaling before amyloid plaque formation prevented the development of synaptic pathology at a later stage in an AD mouse model (Cavanagh et al., 2016). Inhibiting TNFα signaling in 1-month old TgCRND8 mice may normalize the increase in synaptic transmission that is also found at this stage. This increase in synaptic transmission in TgCRND8 mice could be detrimental to synaptic function at later stages, since normalizing the increase by inhibiting TNFα prevented the development of synaptic deficits at advanced stages.

Findings from Cavanagh and colleagues support a hypothesis that increased hippocampal activity is an early-stage change in AD. Previous findings in other APP mouse models have revealed increased neuronal activation near diffuse plaques (Busche et al., 2008) and aberrant electroencephalogram activity (Palop et al., 2007). These functional changes could be related to the increased risk of seizures in AD patients (Pandis and Scarmeas, 2012), especially in cohorts of early-onset and familial AD (Cabrejo et al., 2006; Scarmeas et al., 2009). In human studies, increased hippocampal activity has been observed in asymptomatic carriers of AD-related genes (Nichols et al., 2012; Reiman et al., 2012). Furthermore, in a longitudinal study, the levels of cortical activity in ApoE4 carriers correlated with future degree of cognitive impairment (Bookheimer et al., 2000), suggesting a causal relationship between a preclinical increase in brain activity and cognitive decline. The increase in synaptic function we observed in young TgCRND8 mice could have a significant impact on the hippocampal network, since measuring hippocampal oscillations of pre-plaque TgCRND8 mice has revealed a decrease in theta-gamma cross frequency coupling (Goutagny et al., 2013). While mechanisms underlying the preclinical increase in brain activity in AD remain unclear, they could be related to alterations in inhibitory synapses. The reduction of GABAergic synaptic function has been suggested to underlie enhanced neuronal activation near amyloid plaques in the frontal cortex (Busche et al., 2008). Moreover, decreased GABAergic transmission resulting from decreased expression of the voltage-gated sodium channel subunit Nav1.1 has been detected in the hAPP-J20 mouse model as well as in AD patients (Verret et al., 2012) and GABAergic neurons have been shown to be vulnerable to Aβ toxicity (Krantic et al., 2012). Although we observed increased evoked excitatory synaptic function in the hippocampal CA1 region of pre-plaque TgCRND8 mice, we found no change in miniature excitatory synaptic transmission (Cavanagh et al., 2016). These findings suggest that alterations at the network level, may be related to a reduction in inhibitory synaptic inputs that could underlie the enhanced evoked synaptic responses we observed in these mice. However, changes in inhibitory function could be compensated after enhanced excitability. In the J20 AD mouse model, seizure activity in the hippocampal region was associated with the sprouting of inhibitory inputs in the dentate gyrus (Palop et al., 2007). Further studies using young asymptomatic APP mouse models are needed to reveal synaptic mechanisms that are responsible for causing neuronal hyperexcitability in APP mouse models.

Before amyloid plaque formation, an increase in inflammatory mediators, such as activated microglia (Wright et al., 2013) and cytokines (Goutagny et al., 2013; Hanzel et al., 2014; Janelsins et al., 2005), has been observed in cortical and hippocampal regions of APP rodent models. Data from our intervention study suggest that TNFα inhibitors may be useful in preventing synaptic deficits in AD. TNFα can enhance neuronal excitability by increasing and decreasing the expression of sodium (Chen et al., 2015) and potassium channels (Diem et al., 2001), respectively. Excitatory synaptic transmission can also be enhanced by TNFα through insertion of calcium permeable GluA2-lacking AMPA receptors (Leonoudakis et al., 2008), which could be mediated by TNFR1 on astrocytes (Habbas et al., 2015). TNFα also enhances the insertion (Wheeler et al., 2009) and calcium influx (Jara et al., 2007) of the NMDA subtype of glutamate receptors. Finally, TNFα can facilitate glutamate release from astrocytes (Santello et al., 2011). Alternatively, activation of neuronal TNFR1 can lead to the endocytosis of GABA_A_ receptors (Pribiag and Stellwagen, 2013). While the pro-excitation effects of TNFα are important for synaptic scaling (Stellwagen and Malenka, 2006), an activity-dependent physiological process for refining neuronal networks, the chronic increase in TNFα we observed in TgCRND8 mice (Cavanagh et al., 2013) could result in hyperexcitability and enhanced sensitivity to excitotoxicity. Increased levels of pro-inflammatory markers like other cytokines (e.g. IFN-γ (Abbas et al., 2002), interleukin1β, interleukin 6 (Heneka et al., 2005)) and the complement system (Hong et al., 2016) have been reported in pre-plaque APP mouse models. Therefore, future studies should find out whether an early-stage increase in these inflammatory markers is related to the alteration of synaptic and neuronal function of APP mouse models.

We found that a 30-day long XPro1595 treatment rescued synaptic deficits in post-plaque TgCRND8 mice 4 months later, suggesting a long-term effect of inhibiting TNFα signaling on preventing synaptic deficits. Synaptic deficits correlate closely to cognitive impairment in AD (Terry et al., 1991) and TNFα inhibitors have been shown to rescue cognitive impairments in APP mouse models (Gabbita et al., 2012; Tweedie et al., 2012). Our findings suggest that inhibiting soluble TNFα signaling alone by XPro1595 is sufficient to prevent synaptic deficits. Notably, while we observed a protective effect of XPro1595 in young TgCRND8 mice, this drug has also been shown to rescue impairments in synaptic function and plasticity in aged rats (Sama et al., 2012), suggesting a protective effect of this drug in both the immature and mature nervous system. Inhibiting TNFα signaling may ameliorate synaptic pathology by preventing the formation of hyperexcitability. Indeed, recent studies using chemogenetic and optogenetic techniques have revealed a causal relationship between brain hyperactivity and the formation of amyloid and synaptic pathologies in APP mice (Yamamoto et al., 2015; Yuan and Grutzendler, 2016). Alternatively, TNFα inhibition could slow down synaptic deficits by interrupting APP processing. Amyloid pathology and Aβ production in APP mouse models can be attenuated by blocking TNFα signaling using either pharmacological or genetic approaches (He et al., 2013, He et al., 2007; Shi et al., 2011) (but also see (Chakrabarty et al., 2011)). TNFα stimulation can increase BACE1 expression and Aβ production in a dose-dependent manner in astrocytic and neuronal cultures, an effect that was blocked by a TNFα-neutralizing antibody (Yamamoto et al., 2007). The impact of TNFα on BACE1 expression may be related to TNFR1-mediated activation of NF-κB, which has binding sites near the BACE1 promoter (Sambamurti et al., 2004). Indeed, TNFα-induced BACE1 promoter activity can be reduced by an NF-κB inhibitor (He et al., 2007).

The effect of XPro1595 on preventing synaptic deficits in an AD mouse model at a pre-plaque stage suggests that inhibiting TNFα signaling can be a preventive treatment for AD. Although recent randomized clinical trials have failed to reveal intervening effects of non-steroidal anti-inflammatory drugs for AD (Heneka et al., 2015), anti-inflammatory drugs could have more success in treating subjects at younger ages (Hayden et al., 2007). Unlike findings from animal studies, in preclinical AD patients, evidence is available both for (Fonseca et al., 2004; Monson et al., 2014) and against (Morimoto et al., 2011) the possible rise of inflammatory markers. Longitudinal studies with bigger sample sizes and unified methodology are therefore needed to find out whether increased levels of TNFα and other pro-inflammatory agents can be found in preclinical AD. A side effect of TNFα inhibitors is immunosuppression and increased susceptibility of infection. Our findings that the protective effect of a short-term treatment (30 days) of a TNFα inhibitor is long lasting suggests that the continuous treatment with a TNFα inhibitor may not be necessary to delay the onset of synaptic deficits. In addition, the new generation of TNFα inhibitors that target soluble TNFα signaling through TNFR1 (e.g. XPro1595) could have lower side effects since the immunomodulatory functions of TNF2 receptor would be spared (Medeiros and LaFerla, 2013).

In conclusion, increased levels of TNFα may be a potential early-stage biomarker in TgCRND8 mice before amyloid plaque formation. Indeed, inhibiting TNFα signaling using the soluble TNFα inhibitor, XPro1595, in young TgCRND8 mice prevented synaptic deficits of these mice at 6 months of age (Cavanagh et al., 2016). These findings suggest that inhibiting TNFα signaling could be an intervention approach to prevent AD. Future studies should focus on determining the expression profiles of TNFα and other pro-inflammatory agents in preclinical AD and developing TNFα inhibitors with fewer side effects.
